# Modern Comprehensive Metabolomic Profiling of Pollen Using Various Analytical Techniques

**DOI:** 10.3390/molecules30051172

**Published:** 2025-03-05

**Authors:** Petra Krejčí, Zbyněk Žingor, Jana Balarynová, Andrea Čevelová, Matěj Tesárek, Petr Smýkal, Petr Bednář

**Affiliations:** 1 Department of Analytical Chemistry, Faculty of Science, Palacký University, 17. listopadu 12, 771 46 Olomouc, Czech Republic; petra.krejci@upol.cz (P.K.); zbynek.zingor@upol.cz (Z.Ž.); andrea.cevelova@upol.cz (A.Č.); matej.tesarek@upol.cz (M.T.); 2 Department of Botany, Faculty of Science, Palacký University, Šlechtitelů 27, 783 71 Olomouc, Czech Republic; jana.balarynova@upol.cz (J.B.); petr.smykal@upol.cz (P.S.)

**Keywords:** atmospheric solids analysis probe, infrared spectrometry, Magnoliophyta, mass spectrometry, matrix-assisted laser desorption/ionization, metabolomics, metabolite profiling, pollen, secondary metabolite, Pinophyta, taxonomic differentiation, ultra-high-performance liquid chromatography

## Abstract

Pollen is a cornerstone of life for plants. Its durability, adaptability, and complex design are the key factors to successful plant reproduction, genetic diversity, and the maintenance of ecosystems. A detailed study of its chemical composition is important to understand the mechanism of pollen–pollinator interactions, pollination processes, and allergic reactions. In this study, a multimodal approach involving Fourier transform infrared spectrometry (FTIR), direct mass spectrometry with an atmospheric solids analysis probe (ASAP), matrix-assisted laser desorption/ionization (MALDI) and ultra-high-performance liquid chromatography–mass spectrometry (UHPLC-MS) was applied for metabolite profiling. ATR-FTIR provided an initial overview of the present metabolite classes. Phenylpropanoid, lipidic, and carbohydrate structures were revealed. The hydrophobic outer layer of pollen was characterized in detail by ASAP-MS profiling, and esters, phytosterols, and terpenoids were observed. Diacyl- and triacylglycerols and carbohydrate structures were identified in MALDI-MS spectra. The MALDI-MS imaging of lipids proved to be helpful during the microscopic characterization of pollen species in their mixture. Polyphenol profiling and the quantification of important secondary metabolites were performed by UHPLC-MS in context with pollen coloration and their antioxidant and antimicrobial properties. The obtained results revealed significant chemical differences among Magnoliophyta and Pinophyta pollen. Additionally, some variations within Magnoliophyta species were observed. The obtained metabolomics data were utilized for pollen differentiation at the taxonomic scale and provided valuable information in relation to pollen interactions during reproduction and its related applications.

## 1. Introduction

Pollen plays a critical role in the reproduction of plants. It is a fine powder made up of microscopic grains that contain the male gametes necessary for plant fertilization. This small and simple structure has a huge impact on the survival and diversity of plant populations, influencing both ecological stability and agricultural productivity [1,2].

Pollen grains are produced in the anthers or male cones in Pinophyta and are essential for plant reproduction [1]. During pollination, pollen must be transferred to a stigma (in Magnoliophyta) or directly to the ovule (in Pinophyta). This process can occur by various agents, such as wind, water, or animals, including insects, birds, and mammals [2]. The successful transfer of pollen ensures the combination of genetic material, resulting in the production of seeds and the continuation of plant life cycles. Contribution to genetic diversity within plant populations is the most important role of pollen. Cross-pollination, where pollen from one plant fertilizes the ovules of another, provides genetic variability [3]. This diversity is crucial for plants to adapt to environmental conditions and improve their resistance to diseases and pathogens. It ensures the long-term survival of species in dynamic ecosystems. Pollen is necessary not only for plants but also for numerous insect species as food (e.g., bees), which take pollen and nectar for their needs. The plant–pollinator relationship is ultimately beneficial to both and is important for the entire ecosystem [2]. Although pollen is vital for plants and insects, it can reduce human life quality due to pollen allergies. During the spring and summer months, plants release large amounts of pollen into the air, especially wind-pollinated plants, such as grasses or trees, which are the primary source of pollen allergies [4]. Pollen from insect-pollinated plants is typically a lesser source of allergies. This pollen is generally heavier, and its concentration in the air is significantly lower [5]. The immune system of an allergic person identifies pollen as a dangerous substance that must be destroyed. Histamines are released for these purposes, which causes symptoms of allergic reaction [6].

Pollen grains are fascinating structures well designed to fulfil their role in the plant reproduction cycle. The single-grain size typically ranges from 10 to 100 µm. Common pollen grains consist of two protective layers (outer and inner) that surround the cell containing the male gametophyte inside [7,8]. Exine is the tough, outermost layer of a pollen grain and is primarily composed of sporopollenin—a polymer (consisting of mostly lipidic compounds, phenylpropanoids, and carotenoids)—one of the most durable biological materials, which is worthy of detailed investigation for its resistance to UV radiation, high temperatures, and microbial attacks [9,10,11]. The surface morphology of exine strongly differs among plant species. It controls pollen grain adhesion to pollinators and helps disperse pollen in plant populations. Apertures localized on the exine surface play a crucial role in pollen germination (pollen tube release) [7]. Their number and position are useful for pollen classification and identification [12,13]. Under exine, a thin and flexible inner layer (intine) is localized and is composed mainly of polysaccharides. This layer surrounds the cell cytoplasm, provides mechanical stability, and plays an essential role during pollen germination [1]. A detailed investigation of the chemical properties of pollen grains is in high demand in many areas of research and practice, but despite the technological development of analytical techniques available, it is relatively difficult to investigate due to the microscopic dimensions and structural complexity. The development of multimodal analytical procedures is, therefore, currently the subject of intensive research.

Nowadays, microscopic analyses (in brightfield, darkfield, or scanning electron microscope) are mostly used for pollen characterization. Vibrational spectroscopic techniques, such as Fourier-transform infrared (FTIR) and Raman spectroscopy, offer (complementary) the possibility for the simple and rapid acquisition of a “biochemical fingerprint” (overall classification of present chemical structures) but without the detailed identification of molecules. Perhaps the main advantages of these spectroscopic techniques are the possibility of virtually non-invasive analysis without the need for sample preparation and the potential for chemical imaging [14,15]. On the other hand, separation techniques, especially in combination with mass spectrometry, i.e., gas chromatography–mass spectrometry (GC-MS) and high-performance liquid chromatography–mass spectrometry (HPLC-MS), provide pollen chemical characterization on the level of particular compounds in a wide scale based on their chemical properties and contents. However, a more complicated (often multistep) sample preparation, including the extraction and/or derivatization of present analytes after proper optimization, is required [16,17,18].

Currently, we are witnessing the advent of direct mass spectrometry, enabling rapid measurements, usually with a little demand for sample preparation, while providing data for relatively detailed structural analysis. In our previous study, we introduced atmospheric solids analysis probe mass spectrometry (ASAP-MS) for the direct microchemical profiling of pollen grains. This approach enables the detailed study of especially low-polar substances with high sensitivity, which, in combination with multivariate statistics, has been proven to be a useful tool for the chemical characterization of pollen species. Moreover, one pollen grain could be analyzed by this technique [19]. Another widely used tool for the classification of biological samples is matrix-assisted laser desorption/ionization mass spectrometry (MALDI-MS), which allows the high-sensitivity analysis of large molecules, such as proteins, peptides, or carbohydrates. Its application in direct pollen analysis is based on the analysis of these molecules for the subsequent classification of studied pollen species [20]. To briefly describe a potential scenario of direct mass spectrometry application, the well-optimized MS method can be used instead of or as a supportive approach for classical palynological expertise. The sample preparation and data evaluation are relatively easy for implementation in botanical or agricultural practice. In cases where even the combination of microscopy and direct mass spectrometry does not allow the pollen sample to be classified reliably enough, it is possible to use a combination of direct mass and vibrational spectrometry and hyphenated techniques (GC-MS, LC-MS).

This study deals with the potential of the combination of direct vibrational and mass spectrometry for the chemical characterization of non-polar compounds (e.g., esters, sterols, and terpenoids) and UHPLC-MS polyphenol profiling/quantification of pollen species in a wide taxonomical scale that, to the best of our knowledge, has not been described in the literature so far. A combination of these techniques provides a deep insight into the metabolite composition in pollen and helps to understand its biological role, protective mechanisms, presence of allergens, and ecological significance.

## 2. Results and Discussion

**Figure A1:**
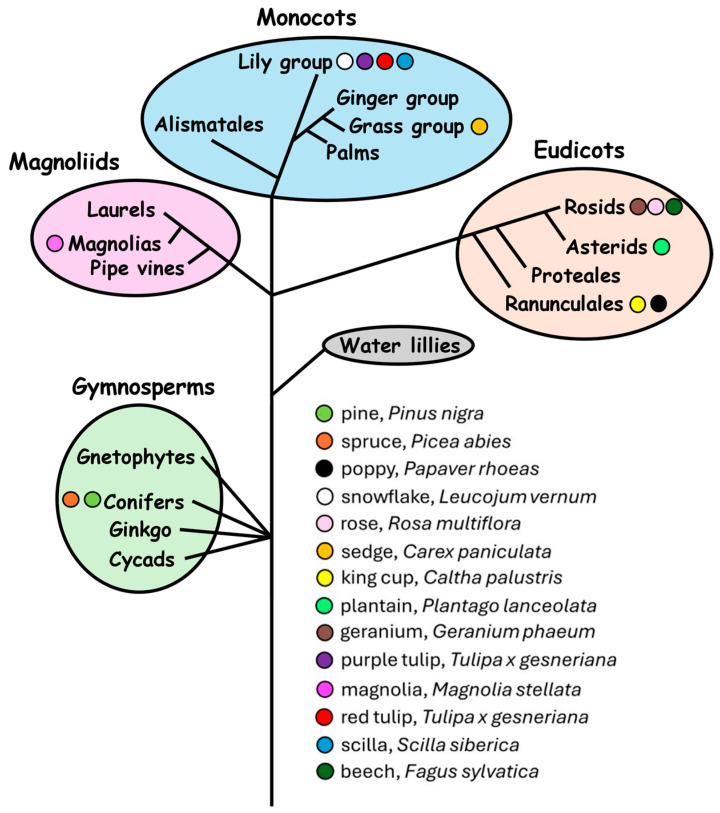
Scheme of taxonomical classification of studied pollen species. Magnoliophyta are currently divided into Liliopsida (monocots), Magnoliopsida (magnoliids), and Rosopsida (eudicots). The latter two involved former dicots.

Pollen from various plant species was subjected to comprehensive chemical analysis based on infrared spectrometry, direct mass spectrometry, and UHPLC-MS. Namely, pollen from pine (*Pinus nigra*), spruce (*Picea abies*), poppy (*Papaver rhoeas*), snowflake (*Leucojum vernum*), rose (*Rosa multiflora*), sedge (*Carex paniculata*), king cup (*Caltha palustrir*), plantain (*Plantago lanceolata*), geranium (*Geranium phaeum*), two different tulips (*Tulip x gesneriana*), magnolia (*Magnolia stellata*), scilla (*Scilla siberica*), and beech (*Fagus sylvatica*) were collected based on taxonomy and its colour. Pinophyta (pine and spruce) and Magnoliophyta involving both monocots (snowflake, tulips, scilla, and sedge) and dicots (magnolia, poppy, king cup, rose, geranium, beech, and plantain) are represented. The detailed classification of studied plant species is schematically described in Figure A1. The various shades of yellow are the most common pollen colouration, but black (red tulip), purple (purple tulip), brown (poppy), and pink (magnolia) are represented among the selected species as well.

### 2.1. Direct Pollen Chemical Characterization

**Figure 1:**
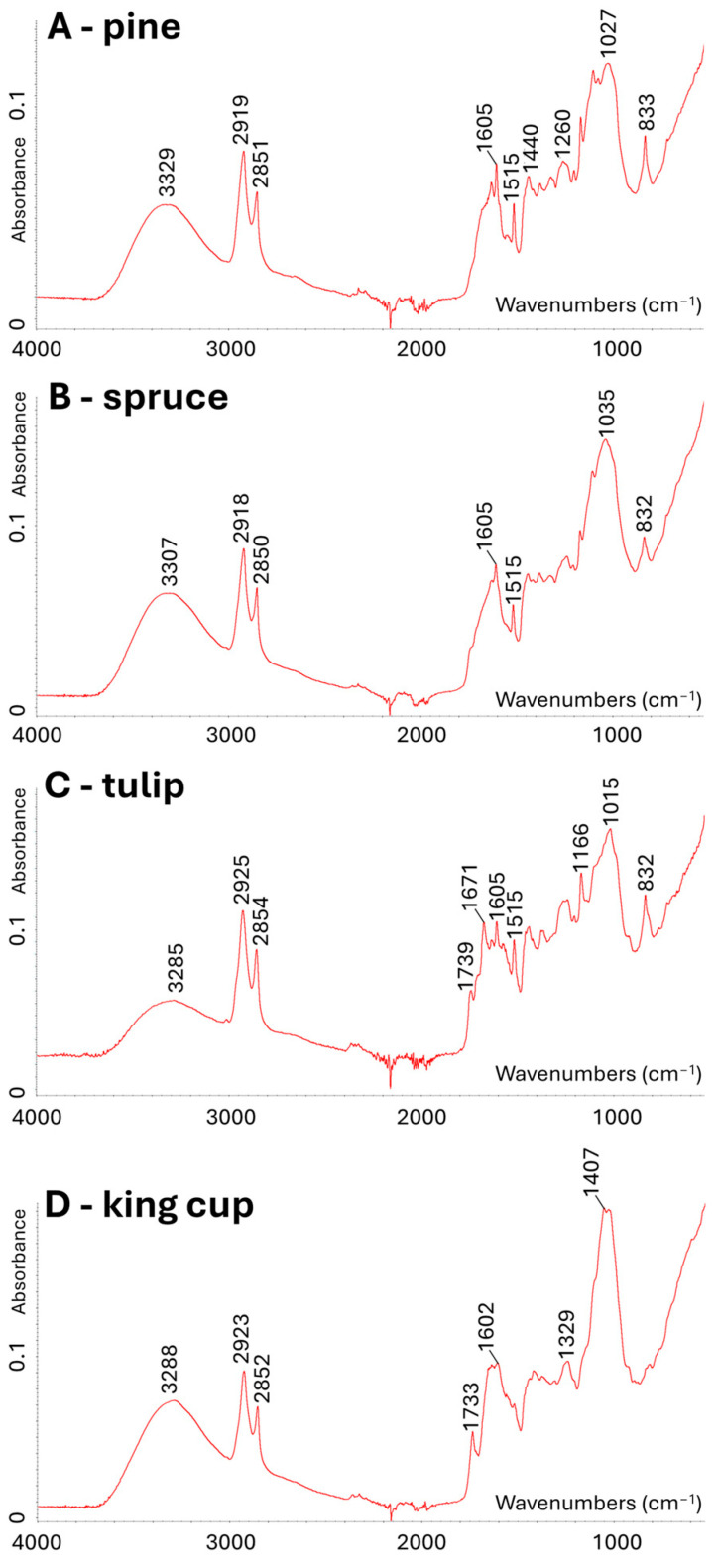
ATR-FTIR spectra of conifer, monocot, and dicot representatives. (**A**)—Pine (*Pinus nigra*). (**B**)—Spruce (*Picea abies*). (**C**)—Tulip (*Tulipa x gesneriana*). (**D**)—King cup (*Caltha palustrir*).

**Figure A2:**
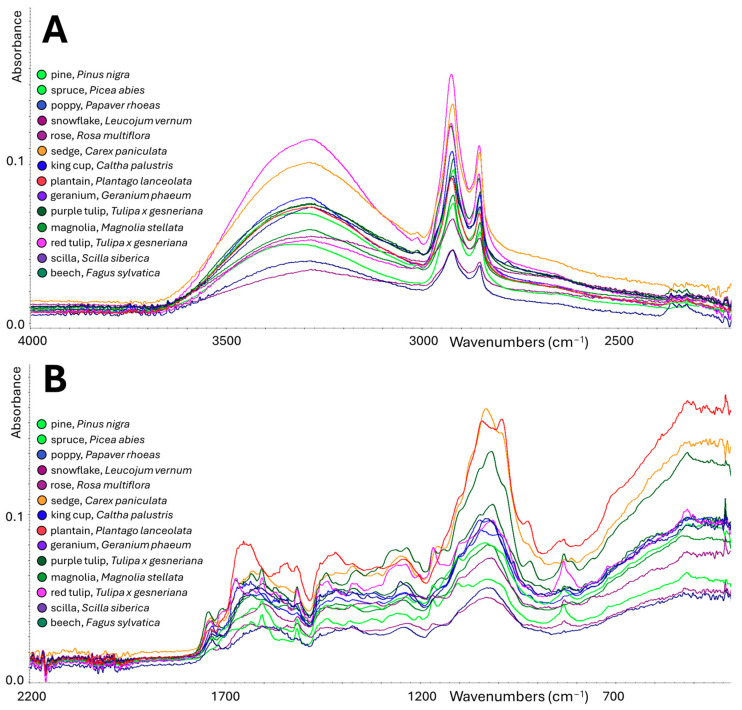
FTIR-ATR spectra of all studied pollen species. (**A**)—Wavenumbers 4000−2200 cm^−1^. (**B**)—Wavenumbers 2200–400 cm^−1^. The top green line represents pine pollen, and the bottom green line represents spruce pollen. The top purple line represents rose pollen, and the bottom purple line represents snowflake pollen. The top dark green line represents magnolia pollen, and the bottom dark green line represents beech pollen. The top dark purple line represents geranium pollen, and the bottom dark purple line represents scilla pollen.

FTIR in the ATR mode was used for the rapid chemical characterization and identification of metabolite groups in pollen species. The vibrational spectra of all pollen species were collected in the wavenumber range 4000–400 cm^−1^ and showed similarities (Figure A2A). In general, three distinct regions can be described, specifically the single-bond region, double-bond region, and fingerprint region. The single-bond region (2500–4000 cm^−1^) is almost identical in shape in all samples. This region contains hydroxyl group stretching (broad band around 3400 cm^−1^), the out-of-phase CH_2_ stretching vibration, and in-phase CH_3_ stretching vibration (a double band around 2920 and 2850 cm^−1^). Minor differences were observed above and below this double band. A small band around 3010 cm^−1^ corresponds to the =C–H stretching vibration and was observed only in the spectra of certain Magnoliophyta species (Figure 1C). In contrast, the signals in the double-bond region showed significant differences (Figure A2B). The presence of lipids is confirmed in FTIR spectra by the first double-bond band of stretching vibrations of the C=O group in alkyl esters (around 1740 cm^−1^). Importantly, this band is completely absent in the spectra of conifers (Figure 1A,B). The most prominent differences were observed under the wavenumber 1700 cm^−1^, respectively, in the fingerprint region. Large variations were observed within the Pinophyta (conifers) and Magnoliophyta species. Furthermore, significant chemical differences were also evident among monocot and dicot species (Figure 1).

Dominant spectral differences are the result of variations in bands associated with sporopollenins, carbohydrates, and esters. Signals around 1605, 1515, 1205, 1170, 855, 830, and 815 cm^−1^ are related to phenyl ring vibrations, and these signals refer to sporopollenin phenylpropanoid structures [14]. Strong carbohydrate signals (C–O, C–C, C–O–C, and C–OH stretches and deformations) were observed in the range 1200–900 cm^−1^. The signals of lipids, i.e., C–O–C stretching in esters, are present at around 1165 cm^−1^ in the FTIR spectra. Specifically, ester compounds (representation and variability) are significantly different within the studied species, as further shown by other techniques. Generally, the fingerprint region is related to skeletal vibrations that are important for accurate identification; nevertheless, it is challenging to interpret. The exact wavenumbers with their respective interpretations have been published in numerous previous studies [11,14,21]. To summarize this part, ATR-FTIR data provided valuable overall insight into the (bio)chemical composition of studied pollens, reflected differences among signals of selected chemical structures and functional groups in relation to plant species on a wide taxonomical scale, and was used as a guideline for consequent detailed mass spectrometric analysis.

**Figure 2:**
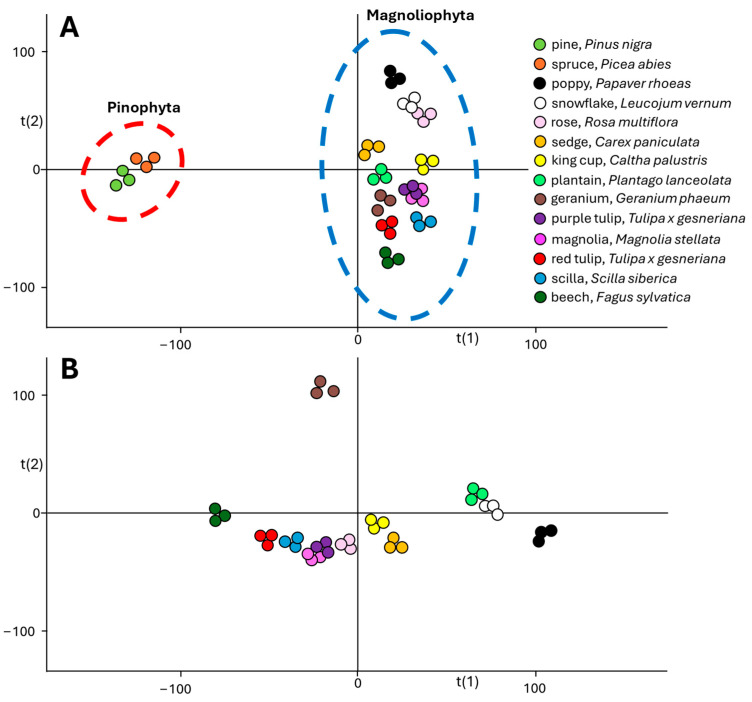
PCA score plot from ASAP-MS analysis in positive ionization mode of studied pollen species. (**A**)—All studied species. (**B**)—Magnoliophyta species.

For ASAP-MS analysis, a small hole was drilled into a standard glass capillary, and pollen samples were inserted inside. The obtained mass spectra were studied using principal component analysis (PCA), and chemical differences were studied. The PCA score plot, shown in Figure 2, revealed a significant chemical diversity between Pinophyta (conifers) and Magnoliophyta representatives.

**Figure 3:**
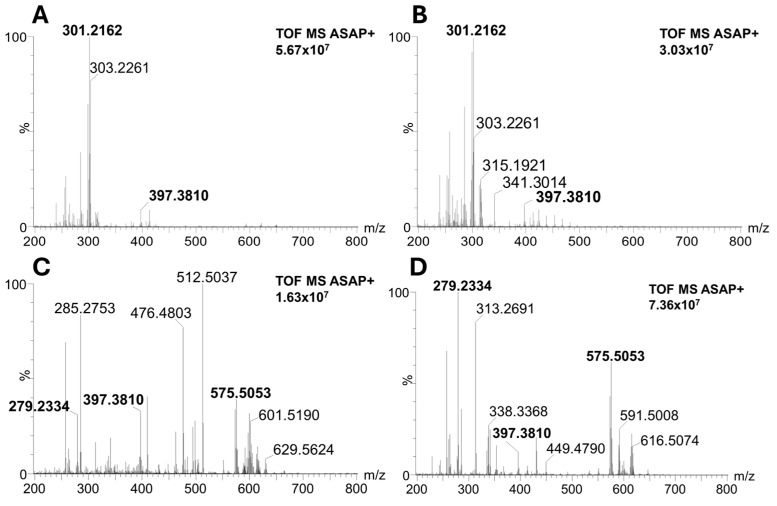
ASAP-MS spectra of conifer, monocot, and dicot representatives. (**A**)—Pine (*Pinus nigra*). (**B**)—Spruce (*Picea abies*). (**C**)—Tulip (*Tulipa x gesneriana*). (**D**)—King cup (*Caltha palustrir*). The intensity number in each panel is referred to as the most intense peak in the spectrum (100%). Identified signals are given in bold.

**Figure 4:**
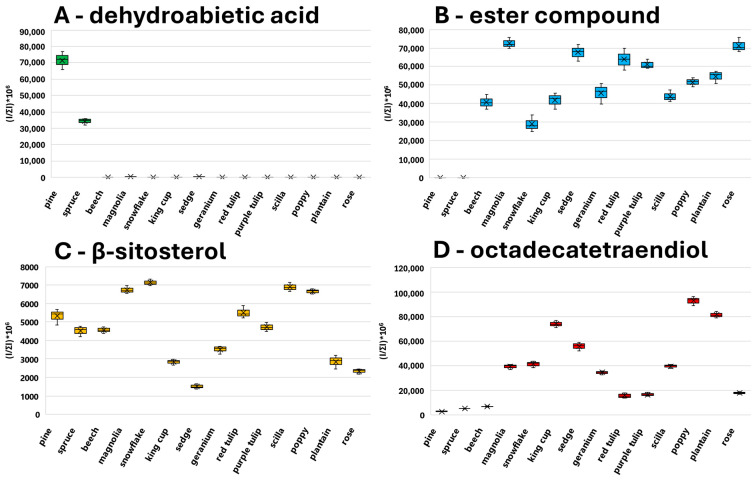
Boxplots of normalized intensities of selected signals in studied pollen species measured by ASAP-MS. (**A**)—Dehydroabietic acid (*m*/*z* 301.2162). (**B**)—Ester of linolenic acid and methyloxooctadecanoate (*m*/*z* 575.5053). (**C**)—β-sitosterol (*m*/*z* 397.3810). (**D**)—Octadecatetraendiol (*m*/*z* 279.2334).

According to the PCA plot, conifer pollen significantly differs from others, and its diversity has been studied in detail. Spectra of conifers (Figure 3A,B) are less abundant in signals above *m*/*z* 500 compared to the spectra of Magnoliophyta (monocots and dicots) species (Figure 3C,D). This fact significantly contributes to the separation of conifers in the PCA plot. Moreover, the characteristic presence of dehydroabietic acid (signal at *m*/*z* 301.2162) was observed in conifer spectra (see boxplots in Figure 4A), and its signal is completely missing in Magnoliophyta pollens (dehydroabietic acid is thus an important marker of conifers). In contrast, abundant signals of esters of fatty acids and alcohols (e.g., ester of linolenic acid and methyloxooctadecanoate, *m*/*z* 575.5053) were observed in Magnoliophyta pollens (Figure 4B) and were not observed in conifer ones (which can be proposed as markers of Magnoliophyta). Other important compounds, like phytosterols (e.g., β-sitosterol, *m*/*z* 397.3810), have been presented with less variability (but provide particular information as discussed later), while certain fatty alcohols (e.g., octadecatetraendiol, *m*/*z* 279.2334) show differences among studied pollen species (Figure 4C,D). The chemical diversity of geranium from other Magnoliophyta species is caused by the presence of specific esters (terpenoids and fatty alcohols), and this is discussed in detail in our previous study [19]. Achieved results, in qualitative accordance with ATR-FTIR data, show the main differences between Pinophyta (conifers) and Magnoliophyta pollens in the presence and content of esters, sterols, and terpenoids. These compounds are part of the hydrophobic barrier on the surface of pollen grains and protect pollen from drying out and exposure to pathogens. Our results show that Magnoliophyta has a hydrophobic barrier that is more chemically diverse and differs from Pinophyta, especially by the presence of various esters. In addition, some structures, such as dehydroabietic acid, significantly contribute to sporopollenin’s antimicrobial protective properties. At the same time, protective compounds could irritate mucous membranes, potentially act as haptens, and play a role in allergic reactions [22]. In a more detailed view, all studied members of the Lily group (snowflake, scilla, and both tulips) provide higher normalized intensities (NIs) of β-sitosterol and lower NIs of octadecatetraendiol. These differences could be potentially used to chemically confirm the presence of Lily group pollen. However, this feature (the useability of these compounds as Lily group markers) needs further proof on a wider range of samples exceeding the scope of this paper. Conifers and monocots (including the Lily group) have a common ancestor among seed plants, which explains their distant evolutionary relationship. A common origin may be the reason for the similar representation of some metabolites in the studied groups of plants, such as conifers and lilies. The identification of all investigated markers was based on exact mass measurement and fragmentation pattern studies. All diagnostic fragments are listed in Appendix B in Table A1.

The ASAP-MS technique was proven to be suitable for the rapid chemical profiling of pollen and its taxonomic classification. Compared to the most used electrospray, low-polar substances can be directly analyzed by mass spectrometry with ASAP ionization. Several fatty acids, long-chain alcohols, terpenes, terpenoids, phytosterols, and various esters were identified abreast in ASAP-MS spectra. In general, the investigation of the lipidic, sterol, and terpenoid profiles could be realized by this approach. We assume that the mentioned metabolite groups come primarily from the exine (the outer layer of the pollen grain), and the developed technique has the potential to reveal the chemical composition of the hydrophobic barrier on the surface of the pollen grain.

**Figure 5:**
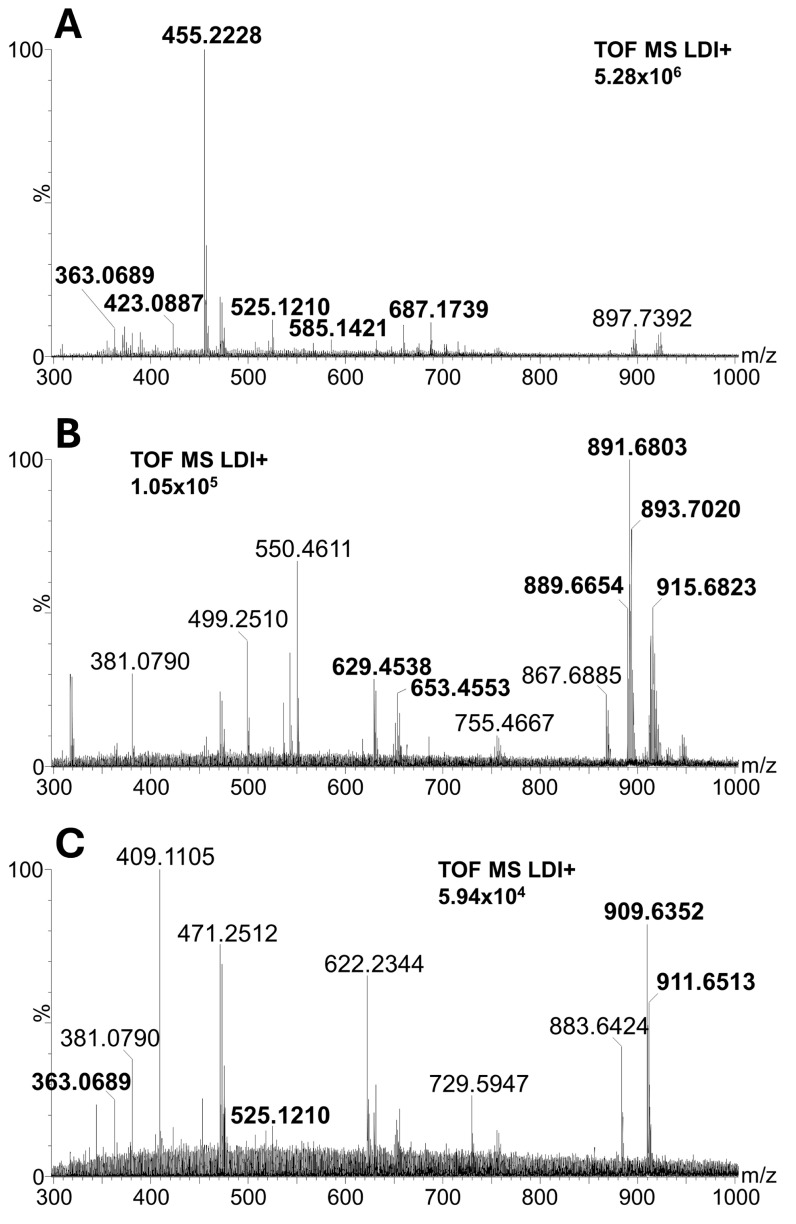
LDI-MS spectra of conifer, monocot, and dicot representatives. (**A**)—Pine (*Pinus nigra*), (**B**)—Tulip (*Tulipa x gesneriana*), (**C**)—King cup (*Caltha palustrir*). The intensity number in each panel refers to the most intense peak in the spectrum (100%). Identified signals are given in bold.

The chemical composition of pollen and its relation to taxonomy was also studied by mass spectrometry with MALDI, which, due to different ionization mechanisms, was complementary to ASAP-MS. During the laser desorption/ionization process, both protective layers of native pollen grains are damaged, and analytes from them and adjacent domains are released. Lipophilic exine, among other functions, protects pollen grains from water to prevent early germination. Observed lipophilic compounds originate dominantly from hydrophobic protective layers. Strong signals of potassium adducts of diacylglycerols (DAG; *m*/*z* 629.4538 and 653.4553) and triacylglycerols (TAG; *m*/*z* 889.6654, 891.6803, 893.7020, 911.6513, and 915.6823) were observed in the spectra of Magnoliophyte species compared to conifers (Figure 5). Polysaccharides (cellulose and hemicellulose) are a major component of the inner layer (intine) and create the solid (reinforcing) structure of the whole pollen grain. Potassium adducts of oligosaccharides (i.e., signals at *m*/*z* 363.0689, 423.0887, 525.1210, 585.1421 and 687.1739) were observed beside signals of lipophilic compounds in MALDI-MS spectra (Figure 5). Structures of lipophilic compounds and oligosaccharides were confirmed by the exact mass measurement and fragmentation pattern study. Diagnostic fragments are listed in Appendix B in Table A2 and in our previous study [23].

**Figure 6:**
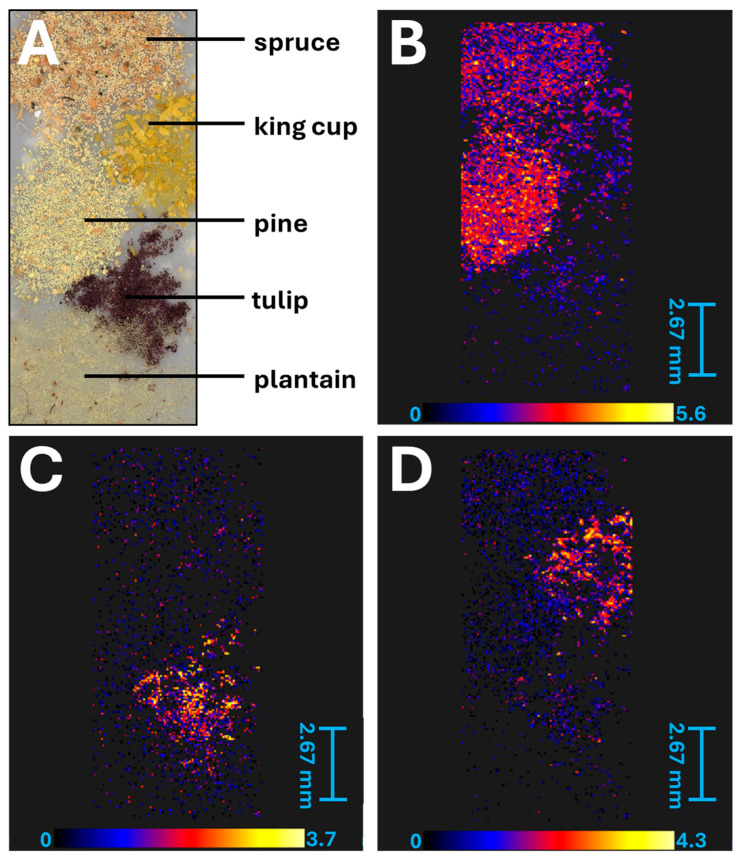
MALDI-MS imaging analysis of selected pollens attached to double-sided tape on the MALDI imaging plate. (**A**)—Photo of the studied pollen mixture. (**B**)—Dehydroabietol cinnamate (*m*/*z* 455.2228). (**C**)—Linolenoyl–linoleoyl–palmitoyl–glycerol (*m*/*z* 891.6803). (**D**)—Trilinolenoyl–glycerol (*m*/*z* 911.6513).

Selected TAGs (*m*/*z* 891.6803 and *m*/*z* 911.6513) were observed in MALDI-MS spectra with different representations in the studied pollen species. These differences were visualized using MALDI-MS imaging and information of the spatial distribution of selected pollen grains was provided (Figure 6). The high relative content of linolenoyl–linoleoyl–palmitoyl–glycerol in tulip pollen and trilinolenoyl–glycerol in king cup pollen allowed for their identification in the pollen mixture. Moreover, the signal with *m*/*z* 455.2228 was identified as a derivate of abietic acid—dehydroabietol cinnamate (in the form of a potassium adduct), and proved to be typical for conifer pollen and, thus, an important marker (Figure 6B). Lipidic and metabolic profiles were found to be characteristic of pollen species and could allow their identification in a mixture.

The MALDI-MS technique is suitable for the direct chemical study of small molecules such as fatty acids, di- and triacylglycerols, and oligosaccharides (from the cell wall). Although many technical aspects should be solved, conceptually, MALDI-MS imaging can be used for the identification of pollen species in a mixture based on their lipidic profile. However, both techniques of direct mass spectrometry (i.e., MALDI-MS and ASAP-MS) appeared in our study to not be suitable for the study of sporopollenin phenylpropanoid structures. A targeted approach and selective analysis based on UHPLC-MS were used for this purpose.

### 2.2. Study of Pollen Polyphenolic Profile

Secondary metabolites, such as phenolic acids and flavonoids, are important products of the phenylpropanoid pathway. These compounds have antioxidant and antimicrobial activity and significantly contribute to the colour and scent of pollen. Visual signals (flavonoids) and scent traces (phenolic acids) belong to key roles of secondary metabolites, thereby increasing pollen attractiveness for pollinators. The UHPLC-MS technique allows the detailed study of polyphenolic profiles in selected pollen species. The UHPLC-MS method and pollen-processing technique were optimized primarily for these purposes.

**Figure 7:**
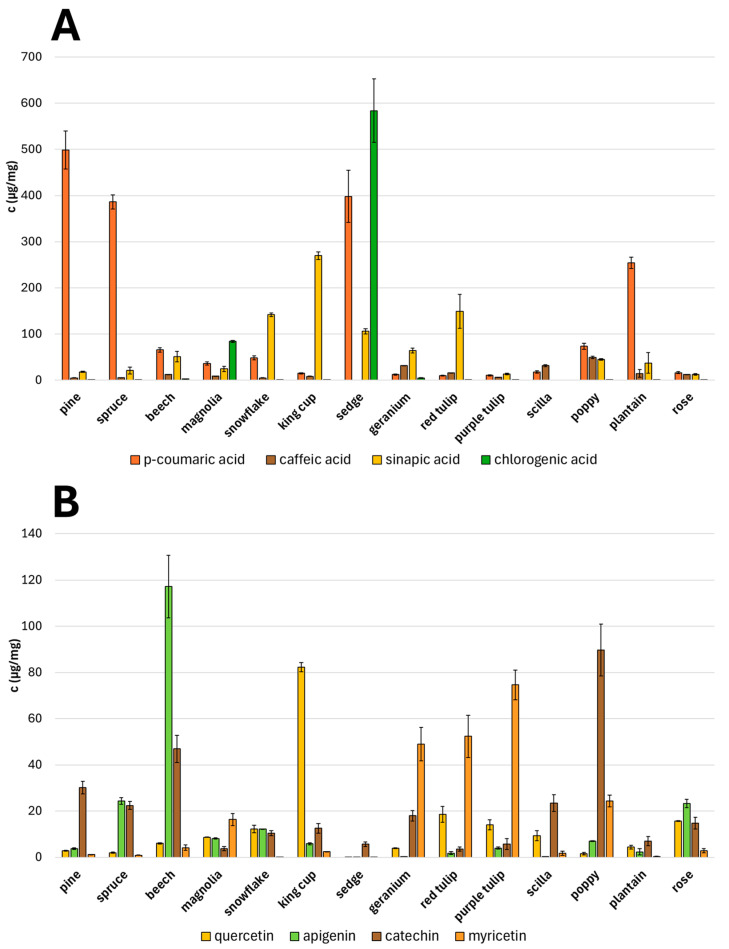
Quantity of selected phenolic acids (**A**) and flavonoids (**B**) in all studied pollen species.

**Figure 8:**
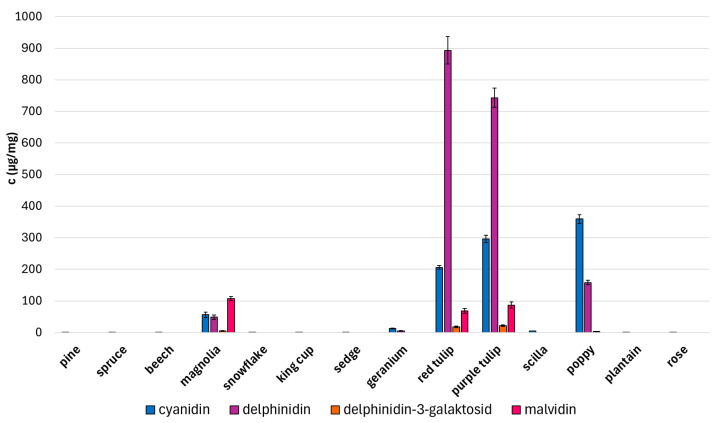
Quantity of selected anthocyanins in studied pollens.

Selected standards of phenolic compounds were analyzed by the UHPLC-MS method, and their characteristic retention times and signals (*m*/*z*) are given in Table 1. Phenolic acids (*p*-coumaric, caffeic, sinapic. and chlorogenic acid) and flavonoids (quercetin, apigenin, catechin, and myricetin) were identified and quantified in all studied pollen species (Figure 7). A huge variability in the content of all phenolic compounds was observed, and different (poly)phenolic profiles are characteristic for each pollen species. In general, the pollen of conifers and grasses is abundant in the content of phenolic acids. A high concentration of these compounds in the air could irritate the mucous membrane and cause an allergic reaction. A high amount of quercetin was found in yellow king cup pollen in contrast with others. The content of selected anthocyanins (cyanidin, delphinidin, delphinidin-3-galactoside, and malvidin) in all studied pollen species is shown in Figure 8. These compounds were observed to have a high content in magnolia (pink), red tulip (black), purple tulip (purple), and poppy (brown) pollens. Only anthocyanins give pollen a different colour from the usual yellow or beige coloration. Moreover, myricetin is described as a copigment occurring together with anthocyanins [24]. Its high concentration was observed in tulip pollens containing a large amount of delphinidin at the same time. The anthocyanin–myricetin interaction could cause the stabilization of the anthocyanin molecules and emphasize pollen colour [25]. Concurrently, all studied compounds provide significant antioxidant and antimicrobial properties for pollen. Pollen grains are, thus, sufficiently protected against external factors such as UV light or pathogens.

**Figure 9:**
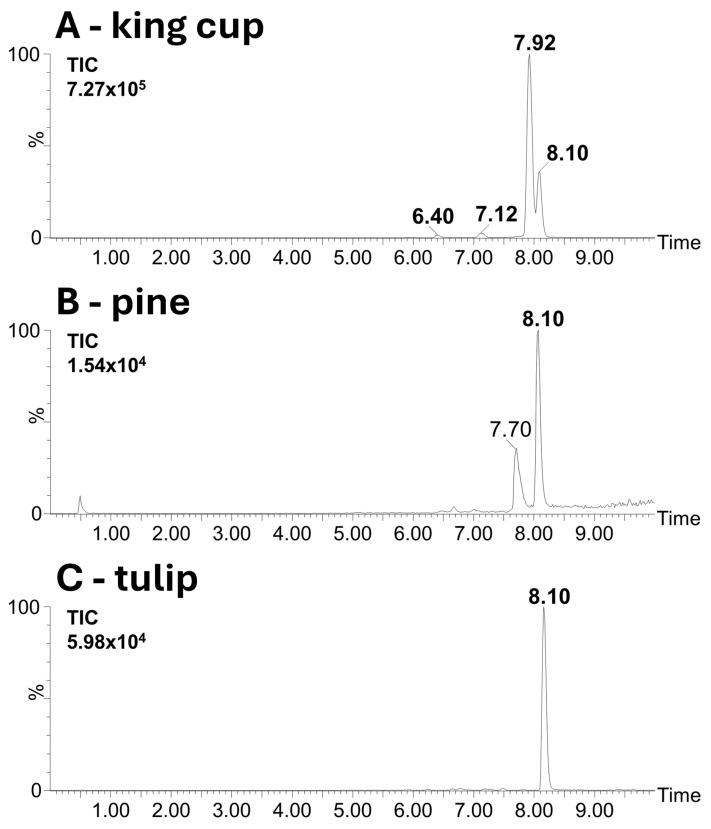
UHPLC-MS analysis of pollen extracts. (**A**)—King cup (*Caltha palustrir*). (**B**)—Pine (*Pinus nigra*). (**C**)—Tulip (*Tulipa x gesneriana*). Chromatograms are reconstructed for *m*/*z* 301.0357 (which represents parent ion of quercetin or aglycone arising during fragmentation of its glycosylated forms). Retention time of peaks of following identified compounds are denoted in bold: quercetin (*m*/*z* 301.0357, Rt 8.10 min), quercetin dihexoside (*m*/*z* 625.1423, Rt 6.40 min), quercetin hexoside (*m*/*z* 463.0886, Rt 7.12 min), and its modified form (*m*/*z* 447.0967, Rt 7.92 min). Intensity is related to most intensive peak in chromatograms (100%). Identified signals are given in bold.

**Figure A3:**
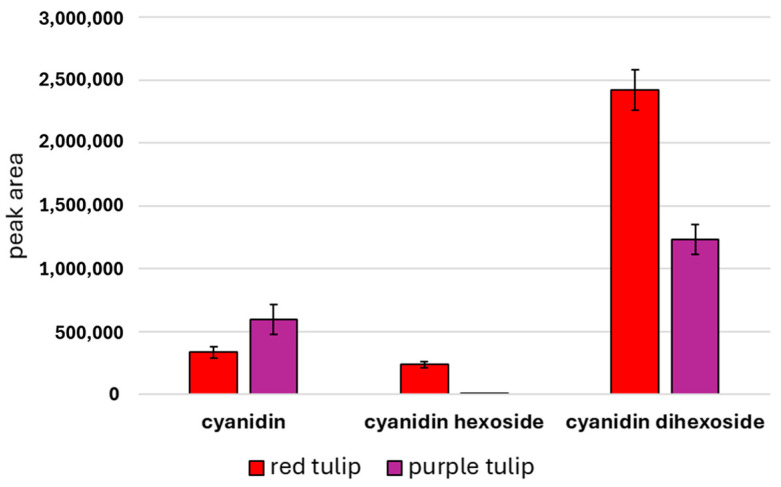
Peak area of cyanidin, cyanidin hexoside, and cyanidin dihexoside in different coloured tulip pollens.

Moreover, polyphenol glycosides were observed and identified in chromatograms. Quercetin dihexoside (*m*/*z* 625.1423, Rt 6.40), quercetin hexoside (*m*/*z* 463.0886, Rt 7.12) and its modified form (*m*/*z* 447.0967, Rt 7.92) were identified in king cup extract in addition to quercetin (*m*/*z* 301.0357, Rt 8.10) (Figure 9A). At the same time, these compounds were not observed in other extracts (Figure 9B,C). The analysis of anthocyanins in tulip pollen allowed us to identify cyanidin dihexoside (*m*/*z* 611.1626, Rt 5.90) and hexoside (*m*/*z* 449.1079, Rt 6.62) as well as cyanidin aglycon (*m*/*z* 287.0568, Rt 7.06). Their different relative contents were observed among red (black pollen) and purple (purple pollen) tulip varieties (Figure A3). Significantly, a higher relative content of cyanidin dihexoside was detected in the pollen of red tulips, while cyanidin aglycon was more widely represented in the pollen of purple tulips. The variable representation of polyphenol derivatives could be one of the main reasons for the different colour of pollen. The identification of compounds was based on exact mass measurements and fragmentation pattern studies. All diagnostic fragments are listed in Appendix B in Table A3.

The introduced UHPLC-MS method with an appropriate sample-processing technique proved to be a key tool for the characterization of polyphenolic profiles in pollen. All polyphenols, i.e., polyphenol monomers and their glycoside forms, were observed with different representations in the studied pollen species and can thus be used for the classification of unknown pollen samples. Pollen coloration is a result of different (poly)phenolic profiles, compound ratios, and their mutual interactions (e.g., oligomer formation). Identified compounds play a role as attractants for pollinators and also have many protective benefits for pollen grains.

**Figure A4:**
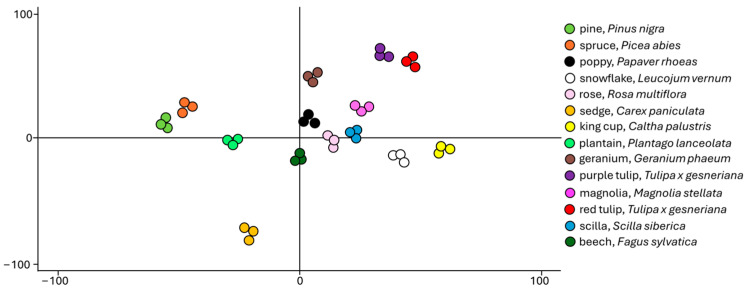
PCA score plot from UHPLC-MS analysis of studied pollen species in negative ionization mode.

The untargeted statistical analysis (PCA) of UHPLC-MS data provided information about the chemical variability of studied pollens. Generally, pollens of wind-pollinated plants (both conifers, sedge and plantain) are localized on the left side of the PCA plot (Figure A4), which indicates their chemically difference nature from insect-pollinated pollens. We believe that UHLC-MS metabolic profiling can be a helpful tool to more deeply understand the mechanism by which pollen disperses in the ecosystem based on, up until now, unknown metabolites. This is an objective for further research.

## 3. Materials and Methods

### 3.1. Chemicals

2-propanol, α-cyano-4-hydroxy-cinnamic acid, acetone, acetonitrile, ethanol, formic acid, leucine-enkephalin, methanol, red phosphorus, and sodium hydroxide were purchased from Sigma Aldrich (St. Louis, MO, USA). Ultrapure water was obtained from the Milli Q apparatus (Merck, Kenilworth, NJ, USA).

### 3.2. Plant Material

**Figure 10:**
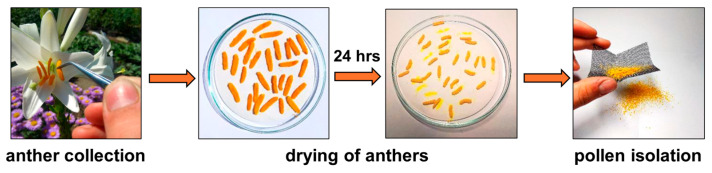
Scheme of another collection and pollen isolation process.

The studied plant material was collected during the spring and summer seasons of 2024 year. The anthers of studied plant species were removed from wild plants in nature using tweezers. Collected anthers were dried for 24 h at room temperature on filter paper in Petri dishes. After drying, pollen grains were separated from anthers using a sifter (mesh size was 0.2 mm). Isolated pollen was transferred into standard plastic microtubes and stored in a freezer (−20 °C). The entire sample collection and its processing are shown in Figure 10.

### 3.3. Sample Preparation

#### 3.3.1. Direct Analysis Using Atmospheric Solids Analysis Probe Mass Spectrometry (ASAP-MS)

Pollen (0.1 mg) was weighed into the modified capillary using analytical balances (Kern, České Budějovice, Czech Republic). The process of capillary modification is described in Section 3.4.1. Glass Capillary Modification. A capillary with pollen was attached to the ASAP and introduced to an ion source for analysis.

#### 3.3.2. Direct Analysis Using Laser Desorption/Ionization Mass Spectrometry (LDI-MS)

Pollen grains were fixed on the MALDI plate (Waters, Milford, MA, USA) using double-sided tape (Ulith, Prague, Czech Republic). The MALDI plate with attached pollen samples was fixed into the holder in the MALDI ion source and introduced to a vacuum chamber for measurements. The solution of α-cyano-4-hydroxy-cinnamic acid (5 mg mL^−1^, dissolved in methanol) was used as a matrix. The matrix was applied to pollen attached on an MALDI plate using a sprayer (SunCollect MALDI Sprayer, SunChrom, Friedrichsdorf, Germany).

#### 3.3.3. Pollen Extracts Analysis Using Ultra-High-Performance Liquid Chromatography Mass Spectrometry (UHPLC-MS)

Pollen (5 mg) was weighed into the plastic microtubes, and the extraction solvent was added (2 mL; acetone/ethanol/methanol/water, 1:1:1:1, *v*/*v/v/v*). Mixtures were sonicated in an ultrasonic bath (Elmasonic, Singen, Germany) for 1 h, shaken overnight using a shaker (UNIMED, Brno, Czech Republic) and centrifugated (Hettich MIKRO 120, Kirchlengern, Germany) for 10 min (14,000 rpm). Supernatants were transferred into glass vials and dried with a fine stream of nitrogen. The obtained residues were dissolved in a mixture of mobile phases (200 µL; A:B, 1:1, *v*/*v*).

### 3.4. Instrumentation

#### 3.4.1. Glass Capillary Modification

**Figure A5:**
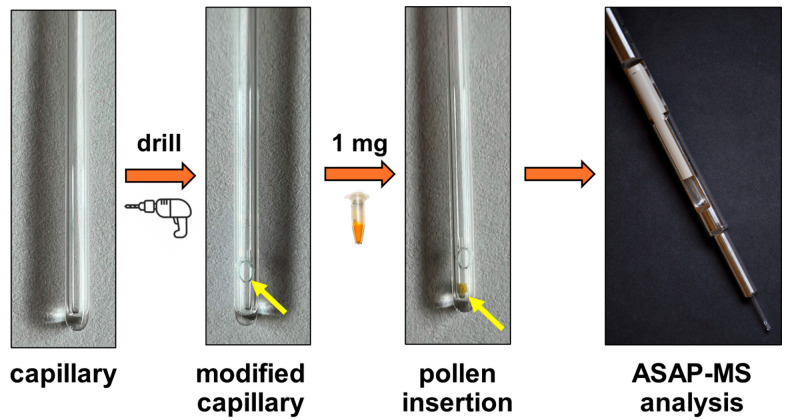
Application of capillary tube for direct pollen analysis using ASAP-MS technique. Arrow shows capillary modification and pollen insertion.

Capillary tubes from soda glass with sealed ends (length 100 mm, Thermo Fisher Scientific, Waltham, MA, USA) were drilled transversely using a diamond drill at a distance of 5 mm from the lower end of the capillary. The drilled hole was used to place pollen into the capillary (Figure A5). A detailed description of capillary tube modifications is given in our previous study [19].

#### 3.4.2. Attenuated Total Reflectance–Fourier Transform Infrared (ATR-FTIR) Spectroscopy

Pollen grains of each sample were measured using a Thermo Scientific™ Nicolet™ iS™50 FTIR spectrometer (Thermo Fisher, Waltham, MA, USA) equipped with a diamond ATR crystal, DTGS detector, and KBr beamsplitter. The spectra were measured in the range of 400 to 4000 cm^−1^ with 4 cm^−1^ resolution, and a minimum of 50 scans were collected from each sample. The background spectrum was collected in advance and automatically corrected.

#### 3.4.3. Atmospheric Solids Analysis Probe Mass Spectrometry (ASAP-MS)

A high-resolution tandem mass spectrometer with an ASAP ion source and QqTOF analyzer with a cyclic ion mobility cell (Select Series Cyclic IMS, Waters, Milford, MA, USA) were used. Mass spectra were collected in the positive ionization mode for 2 min after inserting the ASAP into the ion source. The mass range was set at 50–1200 Da, and the scan time was 1 s. ASAP source parameters were set as follows: cone voltage at 30 V, Source Offset Voltage at 10 V, Source Temperature at 100 °C, Desolvation Temperature at 600 °C, Cone Gas Flow at 30 L h^−1^, desolvation gas flow at 500 L h^−1^, Nebulizer Gas Pressure at 6 Bar, Reference Capillary Voltage at 3.5 kV, and Corona Current at 2 µA. Collision energies were set as follows: Trap Collision Energy (TrapCE) at 6 eV and Transfer Collision Energy (TransferCE) at 4 eV. TrapCE was set at 40 eV, and LM resolution was set at 14.0 (arbitrary unit) for MS/MS experiments. Instrument calibration was performed using sodium formate solution (0.5 mmol L^−1^, dissolved in 90:10 2 propanol/water). Leucine-enkephalin (50 pg µL^−1^, dissolved in 50:50 acetonitrile/water + 0.1% formic acid) was used for lock mass correction using a reference electrospray probe. Lock mass spectra were collected at the end of the analysis for 10 s. Instrument control and data collection were performed using the software MassLynx 4.2 (Waters, Milford, MA, USA).

#### 3.4.4. Matrix-Assisted Laser Desorption/Ionization Mass Spectrometry (MALDI-MS)

A high-resolution tandem mass spectrometer with the vacuum MALDI ion source and QqTOF analyzer with ion mobility cells (Synapt G2-S, Waters, Milford, MA, USA) was used. Ionization in the MALDI ion source was performed by 350 nm of a 1 kHz Nd:YAG solid-state laser. Mass spectra were collected for 3 min in the positive ionization mode using a laser beam (size 60 µm in diameter, laser energy 350 arb). The mass range was set at 50–1200 Da, and the scan time was 1 s. MALDI source parameters were set as follows: extraction voltage at 10 V, Hexapole Bias at 10 V and Aperture at 5 V. Collision energies were set as follows: Trap Collision Energy (TrapCE) at 4 eV and Transfer Collision Energy (TransferCE) at 2 eV. TrapCE was set at 30 eV, and LM resolution was set at 10 for MS/MS experiments. Instrument calibration was performed using red phosphorus (1 mg mL^−1^, suspension in acetone). Red phosphorus was also used for lock mass correction for exact mass measurements. Lock mass spectra were collected at the beginning of each analysis for 20 s. Instrument control and data collection were performed using the software MassLynx 4.1 (Waters, Milford, MA, USA).

#### 3.4.5. Matrix-Assisted Laser Desorption/Ionization Mass Spectrometry Imaging (MALDI-MSI)

The MALDI-MSI technique was used for the imaging of chemical differences and the spatial localization of individual pollens in their mixture. Experimental parameters were set up, and data collection was driven by HDImaging 1.5 software (Waters, Milford, MA, USA). The laser beam size was 60 μm. Spectra were collected in the positive ionization mode with laser energy at 350 arb. The laser repetition rate was set up at 1000 Hz. The mass range was 100–1200 Da. Scan time was set at 1 s, and the number of the most intense peaks was set at 100,000 to decrease the amount of datapoints. The pixel size was set at 65 μm.

#### 3.4.6. Ultra-High-Performance Liquid Chromatography Mass Spectrometry (UHPLC-MS)

The system for Ultra-High-Performance Liquid Chromatography (ACQUITY, Waters, Milford, MA, USA) with mass spectrometry detection (Select Series Cyclic IMS, Waters, Milford, MA, USA) was used. Mobile phase A consisted of water with 0.1% formic acid, and mobile phase B consisted of methanol with 0.1% formic acid. The flow rate of the mobile phase was set at 0.5 mL/min, the time of analysis was 10 min, and the parameters of linear gradient elution were as follows: 0.1% of mobile phase B at the initial time and 100% B at the time 9.00. The injection volume of the sample was 5 µL. Sample separation was realized using the ZORBAX Eclipse Plus C18 column (Agilent, Santa Clara, CA, USA) thermostated at 30 °C. Mass spectrometry detection was performed in the negative ionization mode at the mass range 50–1200 Da. The parameters of the mass spectrometer were as follows: spray voltage 2.5 kV, cone voltage 25 V, desolvation gas flow 600 L/hod, and desolvation gas temperature 220 °C. Instrument control and data collection were performed using the software MassLynx 4.2 (Waters, Milford, MA, USA).

### 3.5. Data Processing

Collected raw MS data were processed by MassLynx software 4.2 (Waters, Milford, MA, USA). The data analysis tool MarkerLynx XS V4.2 was used for data processing, which included data extraction, normalization, and alignment to create the data matrix. MarkerLynx parameters were set as follows for ASAP-MS data: Marker Intensity Threshold at 1000, Peak Separation at 0.05 Da, and mass range at 100–1200 Da. Combined scan range types of analysis were selected. Data sets were analyzed by multivariate statistics software EZinfo (version 3.0, Umetrics, Umeå, Sweden). Principal component analysis (PCA) was used to study the diversity of plant pollen species. Normalized intensities of signals at particular *m*/*z* values were used for boxplot creation. Normalized intensities were calculated by dividing the intensity of the signal of a particular analyte (I) by the sum of intensities of all signals in the studied MS spectrum (ΣI). Collected raw chromatograms were also processed by MassLynx software 4.2. Chromatographic peaks of signals at particular *m*/*z* values were generated and integrated automatically using the software. The obtained peak areas were used to calculate the number of selected compounds. The quantification of selected phenolic acids, flavonoids and anthocyanins was realized using standard calibration curves (Table A4).

## 4. Conclusions

A combination of vibrational (ATR-FTIR) and direct mass (ASAP-MS and MALDI-MS) spectrometry and the hyphenated UHPLC-MS technique provided comprehensive information on the composition of pollen metabolites. This approach allows the comprehensive and synergistic investigation of various metabolite groups in a complex pollen matrix. ATR-FTIR revealed the presence of phenylpropanoid structures, lipids, and carbohydrates. ASAP-MS provided a detailed profile of low-polar compounds such as fatty acids, phytosterols, and terpenoids. In addition, dehydroabietic acid appeared to be an excellent marker of pollen from conifers. This compound is known as an allergen. MALDI-MS revealed the presence of diacyl- and triacylglycerols and oligosaccharide chains. MALDI-MS imaging proved to be helpful during the microscopic tracing of pollen species in their mixture. Finally, UHPLC-MS allowed the detailed profiling of phenolic acids, flavonoids, and anthocyanins. A combination of developed analytical techniques allows the classification of pollen samples in context with their botanical origin. The detected metabolites are important in relation to mechanisms of pollen grain protection, pollen–pollinator interactions, allergic reactions, and the utilization of pollen in various applications.

## Figures and Tables

**Table 1:** Selected standards of phenolic compounds and their characteristic retention times and *m*/*z* signals (in negative ionization mode for phenolic acids and flavonoids and in the positive ionization mode for anthocyanins).

Phenolic Compounds	Retention Time (min)	Signal (*m*/*z*)
*p*-coumaric acid	6.47	163.0402 [M − H]^−^
caffeic acid	5.75	179.0354 [M − H]^−^
sinapic acid	6.62	223.0617 [M − H]^−^
chlorogenic acid	5.46	353.0888 [M − H]^−^
quercetin	8.10	301.0357 [M − H]^−^
apigenin	8.58	269.0468 [M − H]^−^
catechin	8.70	289.0703 [M − H]^−^
myricetin	7.38	317.0290 [M − H]^−^
cyanidin	7.06	287.0568 [M + H]^+^
delphinidin	6.81	303.0517 [M + H]^+^
delphinidin-3-galactoside	5.78	465.1042 [M + H]^+^
malvidin	6.93	331.0824 [M + H]^+^

## Data Availability

The data set is openly available at Zenodo repository (https://zenodo.org/, accessed on 2 March 2025; https://doi.org/10.5281/zenodo.14831672).

## Abbreviations

The following abbreviations are used in this manuscript:

ASAP	Atmospheric solids analysis probe
ATR	Attenuated total reflectance
FTIR	Fourier transform infrared spectrometry
GC-MS	Gas chromatography–mass spectrometry
HPLC-MS	High-performance liquid chromatography–-mass spectrometry
MALDI	Matrix-assisted laser desorption/ionization
NI	Normalized intensity
PCA	Principal component analysis
TAG	Triacylglycerols
UHPLC-MS	Ultra-high-performance liquid chromatography–mass spectrometry

## Appendix B

### Appendix B.1

**Table A1:** List of identified compounds from ASAP-MS analysis of pollens.

Identified Compounds	Signal (*m*/*z*) [M + H]^+^	Diagnostic Fragments (*m*/*z*)	Fragmentation Process
Octadecatetraendiol	279.2334	261.2225	Loss of H_2_O
		243.2119	Double loss of H_2_O
		219.1759	Loss of propanol
Dehydroabietic acid	301.2162	255.2123	Loss of CO_2_
		185.1346	Cleavage of A ring
		173.1333	Cleavage of A ring
		159.1170	RDA cleavage
		133.1003	Cleavage of B ring
β-sitosterol	397.3810	299.2729	22–23 cleavage
		215.1750	Cleavage of D ring
		161.1284	Cleavage of C ring
Ester of linolenic acid and methyloxooctadecanoate	575.5053	367.2859	Loss of pentadecedien
		313.2757	Ester bond cleavage
		263.2390	Ester bond cleavage

### Appendix B.2

**Table A2:** List of identified compounds from MALDI-MS analysis of pollens.

Identified Compound	Signal (*m*/*z*) [M + K]^+^	Diagnostic Fragments (*m*/*z*)	Fragmentation Process
Dehydroabietol cinnamate	455.2228	309.1969	Loss of cinnamate
		265.1305	Combined loss of cinnamate and propane
Linolenoyl–palmitoleoyl–glycerol	629.4538	373.2178	Loss of palmitic acid
		351.2341	Loss of linolenic acid
Linoleoyl–linolenoyl–glycerol	653.4553	375.2345	Loss of linolenic acid
		373.2186	Loss of linoleic acid
Dilinolenoyl–palmitoleoyl-glycerol	889.6654	633.4302	Loss of palmitic acid
		611.4456	Loss of linolenic acid
		333.2218	Loss of two molecules of linolenic acid
Linolenoyl–linoleoyl–palmitoyl–glycerol	891.6803	635.4489	Loss of palmitic acid
		613.4613	Loss of linolenic acid
		611.4462	Loss of linoleic acid
Dilinoleoyl–palmitoleoyl–glycerol	893.7020	637.4612	Loss of palmitic acid
		613.4615	Loss of linoleic acid
Trilinolenoyl–glycerol	911.6513	633.4302	Loss of linolenic acid
		355.2069	Loss of two molecules of linolenic acid
Dilinoleoyl–linolenoyl–glycerol	915.6823	637.4618	Loss of linolenic acid
		635.4468	Loss of linoleic acid

### Appendix B.3

**Table A3:** List of identified compounds from UHPLC-MS analysis of pollens.

Identified Compounds	Signal (*m*/*z*) [M − H]^−^	Diagnostic Fragments (*m*/*z*)	Fragmentation Process
quercetin hexoside (modified)	447.0967	301.0357	Loss of deoxy-hexose
quercetin hexoside	463.0886	301.0357	Loss of hexose
quercetin dihexoside	625.1423	445.0892	Loss of hexose
		301.0357	Loss of two molecules of hexose
**Identified Compounds**	**Signal (*m*/*z*) [M + H]^+^**	**Diagnostic Fragments (*m*/*z*)**	**Fragmentation Process**
cyanidin hexoside	449.1079	287.0568	Loss of hexose
cyanidin dihexoside	611.1626	431.0992	Loss of hexose
		287.0568	Loss of two molecules of hexose

### Appendix B.4

**Table A4:** Calibration dependencies of selected phenolic acids, flavonoids, and anthocyanins.

Phenolic Compound	Regression Function	Reliability Value (R^2^)
*p*-coumaric acid	y = 116,795x + 280.6	0.9998
caffeic acid	y = 22,170x − 205.7	0.9999
sinapic acid	y = 71,852x + 4.8148	0.9999
chlorogenic acid	y = 30,942x + 533.15	0.9971
quercetin	y = 513,291x − 689.74	0.9993
apigenin	y = 374,501x − 2487.1	0.9998
catechin	y = 47,518x + 0.2965	0.9999
myricetin	y = 45,976x + 9.5556	0.9998
cyanidin	y = 111,249x − 1682.1	0.9998
delphinidin	y = 360,858x + 7031.8	0.9997
delphinidin-3-galactoside	y = 91,691x + 96.52	0.9995
malvidin	y = 869,182x − 4195.32	0.9979
